# Usefulness of peak frequency in Electrograms for elimination of left atrial Posterior Wall residual potentials via Epicardial connections

**DOI:** 10.1002/joa3.13183

**Published:** 2024-11-19

**Authors:** Masahiro Ishikura, Yoshiaki Kawase, Hiroki Kamiya, Taiji Miyake, Hitoshi Matsuo

**Affiliations:** ^1^ Department of Cardiology Gifu Heart Center Gifu Japan

**Keywords:** atrial fibrillation, catheter ablation, epicardial connection, left atrial posterior wall

## Abstract

Left atrial posterior wall (LAPW) residual potentials via epicardial connections during LAPW isolation are often low‐frequency continuous potentials. Accurate annotation of these residual potentials by three‐dimensional electroanatomical mapping systems is challenging and can be misleading. Herein, we present a case of successful LAPW isolation using peak frequency analysis.

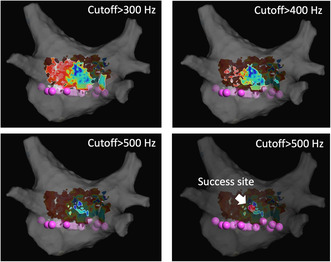

1

Circumferential pulmonary vein isolation (PVI) is a widely accepted and effective therapy for atrial fibrillation (AF). However, AF recurrence rates remain high, particularly in persistent AF.^1^ In addition to PVI, several strategies have been investigated to reduce AF recurrence. Among these, left atrial posterior wall (LAPW) isolation has a clear endpoint of elimination of LAPW potentials and is widely performed in the expectation of additional effect.^1^ However, achieving complete LAPW isolation can be challenging due to the presence of epicardial connections (ECs). Residual potentials via ECs are low‐voltage continuous potentials, automatic electrograms (EGMs) annotation in three‐dimensional electroanatomical mapping systems is often difficult to annotate accurately and can be misleading.^2^ In addition, extensive ablation of the LAPW increases the risk of esophageal injury. Here we present a case of successful LAPW isolation using peak frequency analysis to eliminate residual potentials via ECs.

A 53‐year‐old man with tachycardia‐induced cardiomyopathy due to persistent AF was referred to our hospital. We performed catheter ablation for persistent AF under the guidance of a three‐dimensional electroanatomical mapping system (EnSite X™ EP system, (Abbott, St. Paul, MN, USA). A novel peak frequency annotation software, EnSite™ OT Near‐Field, was applied to the local activation time (LAT) map. The pre‐voltage map was obtained using an HD Grid™ SE mapping catheter (Abbott) (Figure 1(A)), revealing no low‐voltage areas in the left atrium (LA). After PVI and a LA roof line ablation using a cryoballoon (ArcticFront Advance Pro, Medtronic Inc.), post‐PVI and roof line voltage map was obtained (Figure 1(B)). Radiofrequency (RF) catheter ablation was then performed to create a bottom line using a TactiFlex™ ablation catheter (Abbott) (50 W for 5–10 s). However, complete isolation of the LAPW was not achieved. The LAT and voltage maps of the LAPW were obtained during pacing from the coronary sinus ostium (Figure 1(C,D); a video file corresponding to Figure 1(C) is also available as supplemental material). Most of the LAPW EGMs were low‐voltage continuous. Inconsistent positioning of the annotation was observed. To confirm that residual potentials of LAPW were not conducting from line gaps, we also obtained a LAT map of the LA during pacing from the LAPW. The earliest activation site (EAS) in the LA was 14 mm away from the bottom line (Figure 2). To determine ablation sites to eliminate residual potentials in the LAPW, we used an emphasis map that, by setting a cutoff value of the peak frequency, can emphasize the areas where the higher peak frequency exists above the cutoff value. As the peak frequency cutoff was increased, the higher peak frequency area was localized to the lower center of the LAPW. (Figure 3). Residual potentials in the LAPW were successfully eliminated after only one RF applications at sites where peak frequencies exceeded 500 Hz. The successful ablation site was far from the EAS site in the LAT map (Figure 4(A)). The EGM at the EAS site was a dull potential with a low peak frequency, indicating that it was a far‐field potential. Two additional RF applications were performed, followed by post‐voltage map, which confirmed extensive isolation of the LAPW (Figure 4(B)).

**FIGURE 1:**
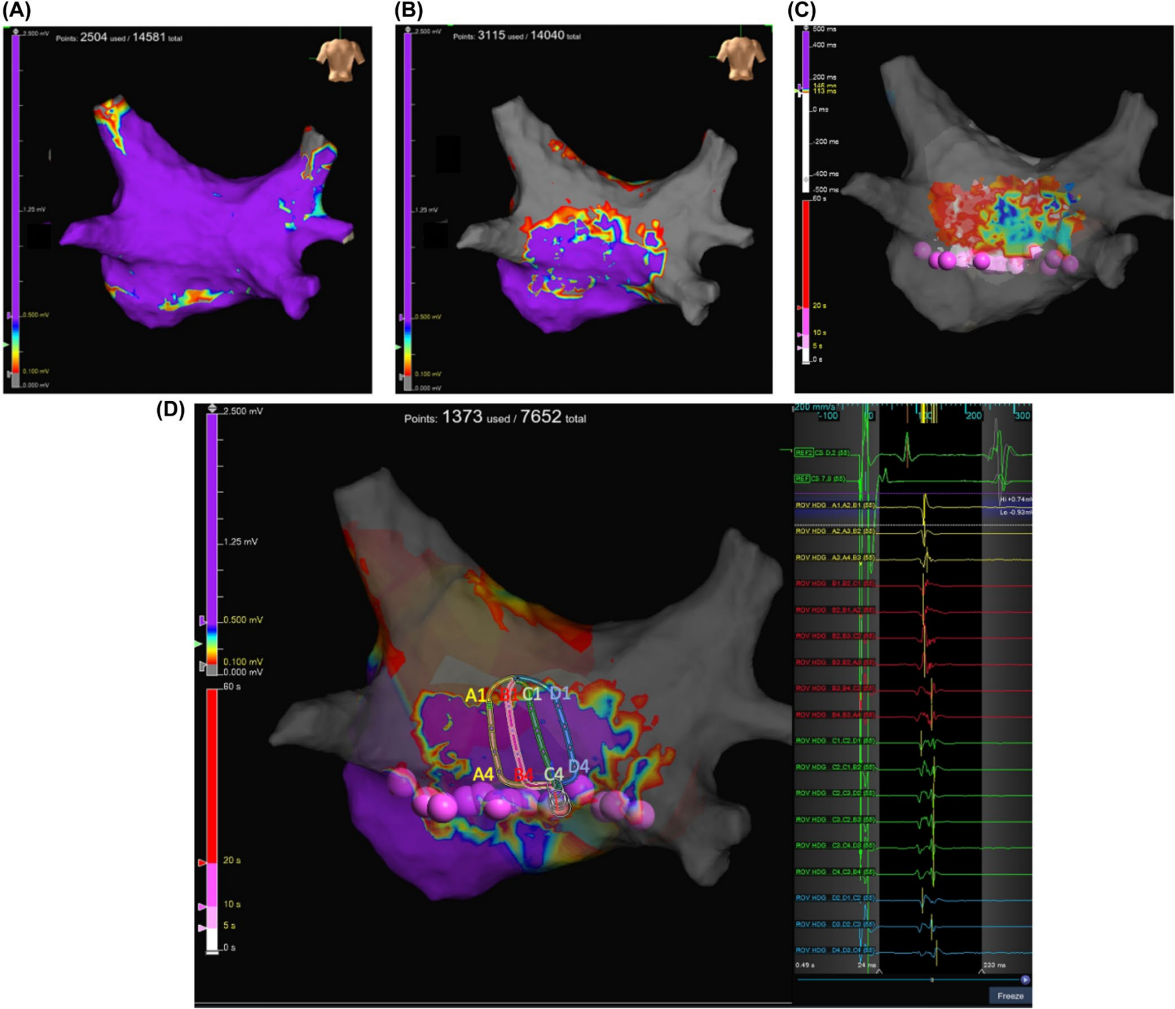
(A) Pre voltage map of the left atrium (LA). (B) Post voltage map of the LA after pulmonary vein isolation and roof line ablation. (C) Local activation time map of residual potentials in left atrial posterior wall (LAPW). (D) Voltage map and local electrograms (EGMs) in the LAPW. Yellow spike in the EGMs indicates automatic annotation.

**FIGURE 2:**
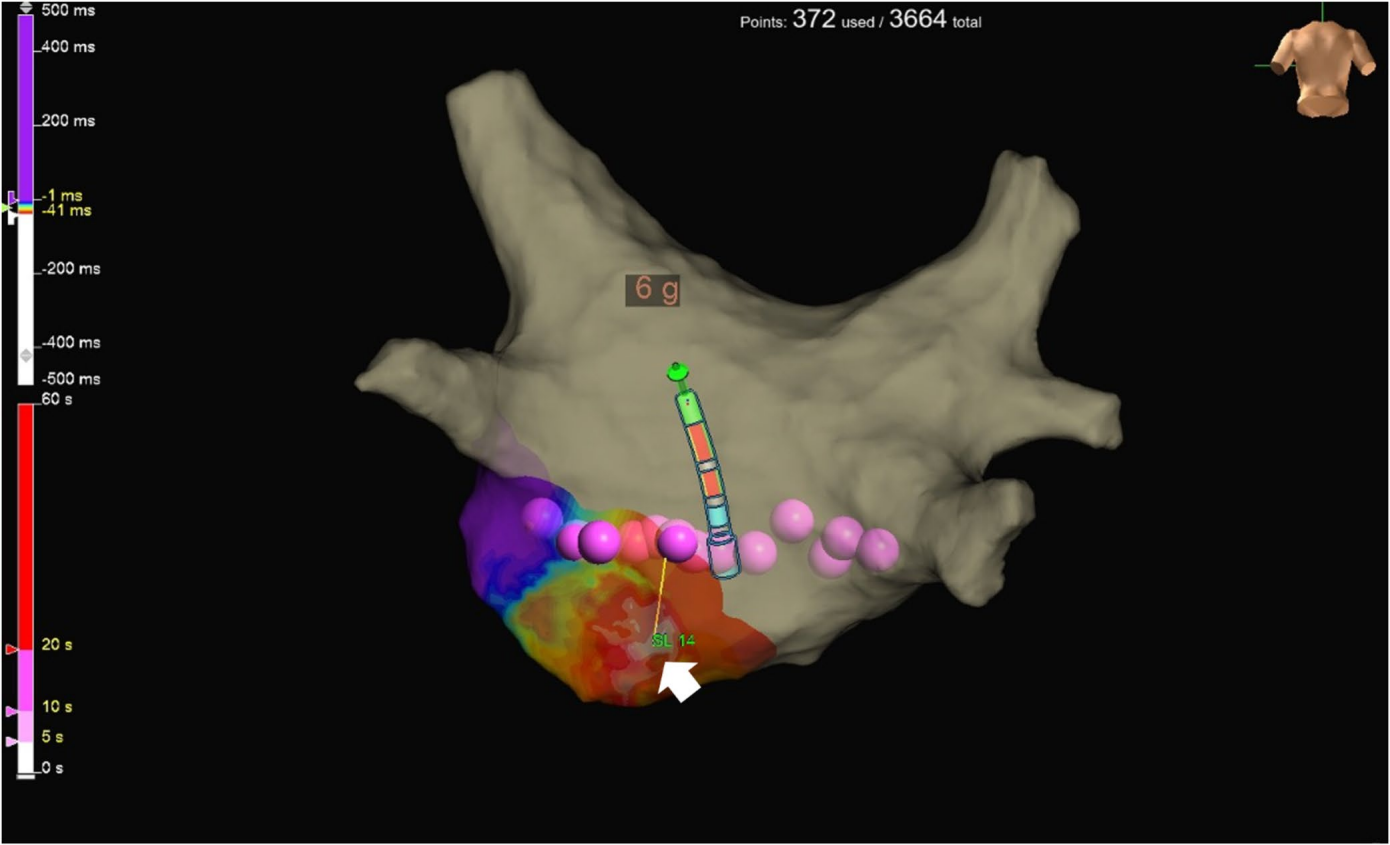
Local activation map of left atrium during pacing from left atrial posterior wall. The earliest activation site (EAS) was 14 mm away from the bottom line. The white arrow indicates the EAS site.

**FIGURE 3:**
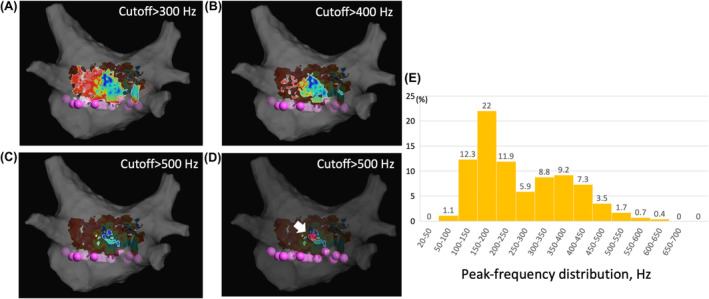
(A) Emphasis map: Cutoff value of peak frequency >300 Hz. (B) Cutoff >400 Hz. (C) Cutoff >500 Hz. (D) Emphasis map (Cutoff >500 Hz) and successful isolation site. White arrow indicates a success tag of the left atrial posterior wall (LAPW) isolation. (E) Peak‐frequency distribution in LAPW.

**FIGURE 4:**
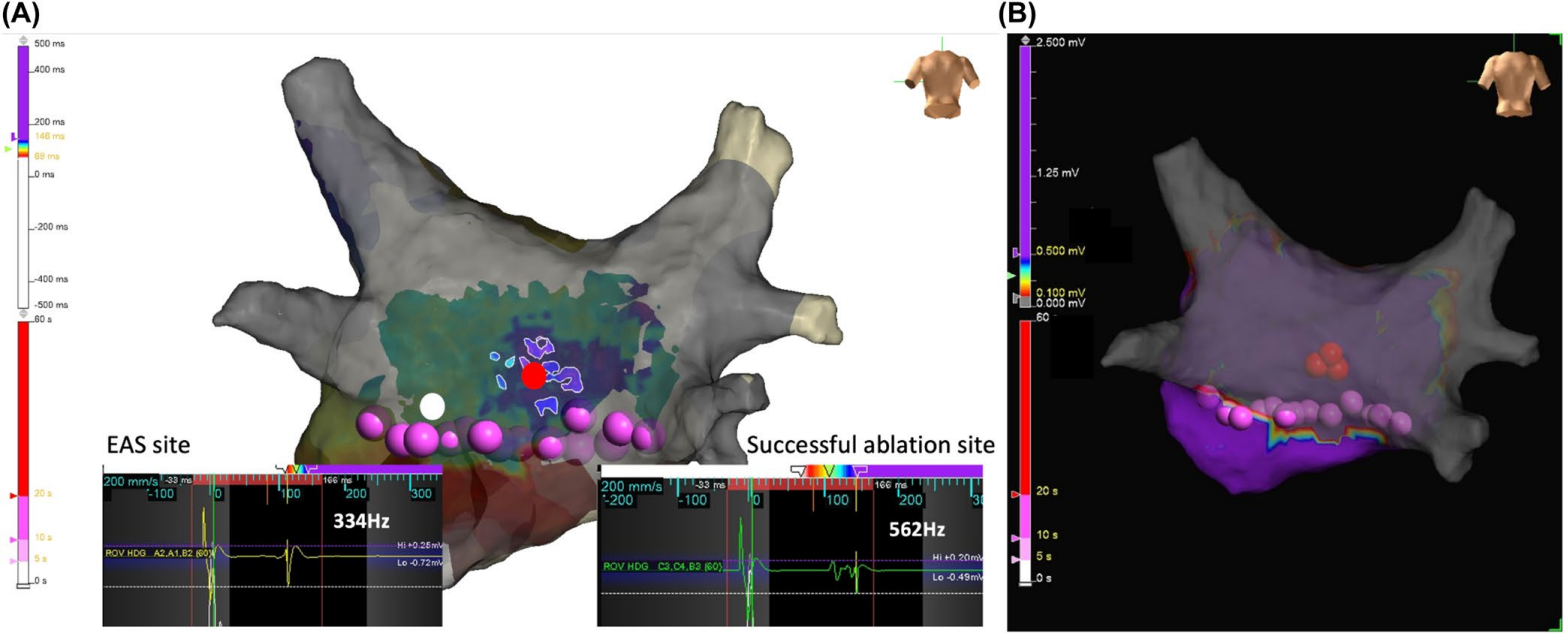
(A) Emphasis map: Cutoff value of peak frequency >500 Hz. The red dot indicates the successful ablation site, and the white dot indicates the earliest activation site (EAS) in the left atrial posterior wall (LAPW). The corresponding local electrograms and peak frequency values are shown. (B) Post‐voltage map after LAPW isolation.

In this case, the residual potentials of the LAPW were successfully eliminated with minimal RF application times using peak frequency analysis. Peak frequency is a novel technology, and to the best of our knowledge, there are no reports of using peak frequency analysis for LAPW isolation to eliminate residual potentials via ECs.

The successful isolation site was far from the EAS site in the LAT map. EnSiteTM OT Near‐Field is a novel algorithm integrated into the EnSite X mapping system. In general, near‐field potentials are sharp and have a higher frequency, while far‐field potentials are dull and have a lower frequency. This algorithm automatically annotates the highest peak frequency associated with EGMs, resulting in accurate near‐field potential annotation. However, the residual potential via ECs appears as a low‐voltage continuous potential, which complicates automatic potential annotation and makes it difficult to identify the direction of conduction. In addition, the OT Near‐Field algorithm can annotate near‐field potentials when both far‐field and near‐field potentials are recorded simultaneously. However, when only far‐field potentials are recorded, the OT Near‐Field algorithm annotates far‐field potentials. The local potential at the EAS site in this case was a dull potential, which may have indicated a far‐field potential of ECs or outside of the LAPW.

The successful isolation site was the higher peak frequency value in the LAPW. High peak frequency EGMs in a low voltage area are known to be electrophysiologically important areas where conduction may be disturbed and manifest as a critical isthmus for scar‐related tachycardia.^3^ Peak frequency is a novel technology, the usefulness of which has not yet been fully validated. In the residual potential of LAPW via ECs, our case study suggests that the higher peak frequency EGMs may represent the area of conduction breakthrough from the epicardial to the endocardial layer.

## 2 FUNDING INFORMATION

The authors have no funding information.

## 3 CONFLICT OF INTEREST STATEMENT

All authors have no conflicts of interest to declare.

## 5 ETHICS STATEMENT

N/A.

## 6 CONSENT

The authors confirm that written consent has been obtained from the patient for the submission and publication of this case report, including images and associated test.

## Supporting information


Video S1.

## Data Availability

None.
